# Experiences and Perspectives of People with Stroke, Carers, and Physiotherapists Toward Telerehabilitation Implementation Following Stroke in Saudi Arabia: A Qualitative Study

**DOI:** 10.3390/healthcare14172877

**Published:** 2026-09-07

**Authors:** Bayan Alwadai, Abigail Hall, Hajar Almoajil, Fahad Alotaibi, Saud Alsaadoon, Maedeh Mansoubi, Helen Dawes

**Affiliations:** 1 Department of Public Health and Sport Sciences, Faculty of Health and Life Sciences, Medical School, University of Exeter, Exeter EX1 2LU, UK; 2 Physical Therapy Department, College of Applied Medical Sciences, Najran University, Najran 11001, Saudi Arabia; 3 Department of Health and Community Sciences, Faculty of Health and Life Sciences, Medical School, University of Exeter, Exeter EX1 2LU, UK; 4 Physical Therapy Department, College of Applied Medical Sciences, Imam Abdulrahman Bin Faisal University, Dammam 34212, Saudi Arabia; 5 Department of Physical Therapy, Rehabilitation Hospital, King Fahad Medical City (KFMC), Riyadh 11525, Saudi Arabia; 6 Department of Rehabilitation Services and Programs, Sultan Bin Abdulaziz Humanitarian City (SBAHC), Riyadh 13571, Saudi Arabia; 7 Digital Health & Applied Tech Assessment, Better Health & Care Hub, Faculty of Nursing, Midwifery & Palliative Care, King’s College London, London SE1 9NH, UK

**Keywords:** stroke, telerehabilitation, physiotherapy, Saudi Arabia, qualitative research

## Abstract

**Background/Objectives**: Recovery of function and mobility after stroke is frequently improved through physical rehabilitation that incorporates multiple therapeutic approaches. However, access to rehabilitation services remains limited globally, particularly for individuals living in rural areas or those with restricted transportation options. These barriers highlight the need to explore technology-enabled approaches to delivering rehabilitation care, including telerehabilitation interventions. This study aimed to explore the experiences and perspectives of people with stroke, their carers, and physiotherapists toward the development and implementation of telerehabilitation interventions after stroke in Saudi Arabia. **Methods**: Semi-structured interviews were conducted with six people with stroke and their carers and ten physiotherapists between March 2025 and July 2025. Purposive sampling was employed, and reflexive thematic analysis was used. **Results**: Three themes were identified. The first theme focused on self-efficacy in the use of telerehabilitation, emphasising the barriers that influence it and what is required to enhance self-efficacy and confidence. The second theme related to motivation to use and engage in telerehabilitation was shaped by various barriers and strategies that promote motivation. The final theme explored cultural aspects and approaches that affect self-efficacy and motivation and support the use and implementation of telerehabilitation interventions in clinical practice. **Conclusions**: Telerehabilitation adoption for people with stroke in Saudi Arabia is influenced by self-efficacy and motivation, which are shaped by cultural factors and barriers such as digital inequity, limited awareness, insufficient training, cultural norms, and preference for in-person care. However, with appropriate training, family involvement, culturally sensitive approaches, and hybrid models, telerehabilitation can support continuity of rehabilitation.

## 1. Introduction

Stroke is a clinically defined syndrome characterised by an acute, focal neurological deficit resulting from vascular injury to the central nervous system, including infarction or haemorrhage [1]. Worldwide, stroke is the third leading cause of disability, after ischaemic heart disease, and the second leading cause of death [2]. Individuals who have had a stroke usually have cognitive, balance, or motor impairments, which can lead to functional dependence and decreased quality of life [3]. Recovery of function and mobility following a stroke is likely enhanced by physical rehabilitation, which combines various therapeutic approaches [4]. However, accessing rehabilitation programs remains challenging, especially for those living in rural areas or with limited access to transportation [5]. Prolonged periods without rehabilitation services may worsen or progress pain and disability among stroke patients [6]. Therefore, to address this situation, it is necessary to consider alternative modes of delivering rehabilitation services [7]. Modern technologies and telerehabilitation have made it possible to provide healthcare services remotely to people who are in need, unable to receive in-person therapy, or affected by crises like the COVID-19 pandemic [5]. Also, since Saudi Arabia’s Vision 2030 initiative aims to enhance healthcare services, encourage the adoption of preventive care measures, attract investment, build a robust healthcare system, and promote healthcare innovation among its stakeholders, telerehabilitation and the integration of advanced technologies into the Saudi healthcare system are essential to achieving these national transformation goals [8].

The clinical effectiveness of telerehabilitation interventions on various outcomes has been demonstrated in controlled trials [9]. However, the extent to which these interventions could be optimised for physiotherapists, people with stroke, and their carers remains unclear. Only a limited number of qualitative studies have explored the experiences and perceptions of stroke patients, their caregivers, and healthcare providers regarding the implementation and acceptability of telerehabilitation interventions after stroke, to identify barriers and facilitators [7,10,11,12,13]. Also, a few quantitative surveys have provided an in-depth exploration of healthcare professionals’ acceptance of and satisfaction with the use of telerehabilitation [14,15]. Findings from these studies generally indicate that healthcare providers report high levels of satisfaction and positive experiences with the use of telerehabilitation. However, these studies were conducted in different contexts and healthcare systems. Contextual dimensions, such as cultural, physical, social, spatial, organisational, political, or economic features of healthcare and the health system, impact the effectiveness and implementation of complex interventions [16]. The UK Medical Research Council (MRC) framework emphasises the importance of developing a theoretical understanding of the processes likely to occur within the intervention, as well as how these processes interact with their context and broader dynamic systems [16].

Therefore, this study aimed to explore the experiences and perspectives of people with stroke, carers, and physiotherapists towards telerehabilitation to inform the development and future implementation of a telerehabilitation intervention for people with stroke in Saudi Arabia. The research question was: What are the experiences and perspectives of people with stroke, carers, and physiotherapists regarding the implementation of telerehabilitation after stroke in Saudi Arabia?

## 2. Materials and Methods

### 2.1. Study Design

This study adopted an exploratory qualitative design. An inductive qualitative approach was used to gain an in-depth understanding of participants’ experiences and perspectives to inform the development and future implementation of a telerehabilitation intervention following stroke in Saudi Arabia. The Consolidated Criteria for Reporting Qualitative Studies (COREQ) checklist [17] was completed (Supplementary Material).

### 2.2. Participants and Recruitment

A purposive sampling method was used to recruit participants representing a variety of demographic characteristics [18] such as age, gender, type of stroke, time since stroke, and varying cognitive and functional abilities by using the Mini-Mental State Examination (MMSE) [19] and Barthel Index (BI) [20]. Health-related quality of life was assessed using the Stroke Specific Quality of Life (SSQoL) [21]. Caregivers’ age, gender, and relationship to patients, as well as physiotherapists’ gender, profession, and years of experience, were also considered. This approach aimed to get a variety of views [22] regarding the implementation of telerehabilitation interventions post-stroke in physiotherapy practice in Saudi Arabia.

The inclusion criteria for participants were: (1) People with stroke (haemorrhagic or ischaemic), aged 18 years or older, with normal verbal communication, in the subacute or chronic phase (more than one month after stroke), living in Saudi Arabia and obtaining a score of seven or eight on the eight decisional capacity questionnaires that are relevant to the consent form content. These requirements are necessary to make sure that people can give consent. The decisional capacity questionnaire’s content is based on the previously utilised University of California Brief Assessment Capacity to Consent (UBACC) questions [23] that have been adjusted for the study’s context (Supplementary Materials). (2) Carers aged 18 years or older for people with stroke, with normal verbal communication and living in Saudi Arabia. (3) Clinical or academic physiotherapists with experience working with neurological conditions, including stroke (at least 2 years of experience) in the KSA. The exclusion criteria were: (1) People with stroke in the acute stage, those diagnosed with other neurological disorders in addition to stroke, or those unable to participate due to severe cognitive impairment [24].

People with stroke and their carers attending the outpatient follow-up appointments at the Rehabilitation Department at King Fahad Medical City (KFMC) and Sultan Bin Abdulaziz Humanitarian City (SBAHC), Kingdom of Saudi Arabia (KSA), who met the eligibility criteria, were invited to participate in the study by the hospitals’ physiotherapists (S.A. and F.A.). The potential physiotherapists were invited by the hospitals’ physiotherapists (S.A. and F.A.) through a verbal invitation. A participant information sheet (PIS) was provided to all potential participants, allowing them sufficient time to review the information before deciding on their participation in the study. A written consent form was given to participants before the interview and was confirmed verbally at the start of the interview. The sample size was informed by information power, which means that the more information the sample has that is relevant to the study objectives, the fewer participants are required [25]. Sufficient information power depends on five key items: study aim, sample specificity, use of established theory, quality of dialogue, and the nature of data analysis [25]. In this study, the research aim was focused on exploring experiences and perspectives towards telerehabilitation following stroke in Saudi Arabia. The sample was specific to this aim and included three relevant stakeholder groups: people with stroke, carers, and physiotherapists, all of whom had direct experience of stroke rehabilitation from different perspectives. Semi-structured interviews generated detailed and relevant accounts of participants’ experiences and perspectives. Reflexive thematic analysis enabled an in-depth, interpretative analysis of patterns of shared meaning across the dataset. Although an established theoretical framework was not used to guide the initial analysis, the focused study aim, sample specificity, quality and relevance of the interview dialogue, and in-depth analytic approach were considered collectively to provide sufficient information power to address the research aim.

### 2.3. Researcher Characteristics and Reflexivity

All interviews were conducted by the first author (B.A.), a registered physiotherapist in the Kingdom of Saudi Arabia (KSA) with clinical experience in neurological rehabilitation, who had no prior relationship with any of the participants. At the time of the study, B.A. was a female PhD student, and her position as a researcher and clinician was considered as part of the reflexive process. Her clinical experience facilitated rapport with participants and supported an understanding of clinical terminology and the cultural and healthcare context in which participants’ experiences were situated. However, this clinical background may also have shaped how questions were phrased, which responses were explored through further probing, and how participants’ accounts of stroke rehabilitation were interpreted. Although B.A. had theoretical knowledge of telerehabilitation developed through research and engagement with the literature, she had limited direct clinical experience in delivering telerehabilitation. Therefore, her position combined insider knowledge of stroke rehabilitation and the Saudi healthcare context with more limited experiential knowledge of telerehabilitation practice.

Throughout data collection and analysis, B.A. remained reflexively aware of how her professional background, theoretical knowledge, and assumptions could shape her engagement with and interpretation of the data. The research team was also involved in the data analysis and included researchers with different professional and cultural backgrounds; not all team members were physiotherapists or from Saudi Arabia. This diversity provided different perspectives that contributed to discussions around the interpretation of the data. Consistent with reflexive thematic analysis, researcher subjectivity was acknowledged as an essential part of the interpretative process rather than something that could be eliminated.

### 2.4. Data Collection

A semi-structured interview was conducted from March 2025 to July 2025. An interview topic guide (Appendix A), which included several open-ended questions, was used to guide the interview with the participant. The guide was developed in collaboration with the study team and the Patient and Public Involvement group (PPI). It was translated into Arabic and tested through PPI to ensure all questions were understandable to people with stroke and carer participants. The questions explored physiotherapists’ experiences and current practices in treating stroke patients, including their views, expectations, challenges, and needs regarding the use of telerehabilitation in clinical practice (Table A1). They also explored people with stroke’s experiences with their condition, rehabilitation, and home exercise programs, as well as carers’ experiences, expectations, challenges, and perceived needs in supporting care and participating in telerehabilitation delivery (Table A2 and Table A3).

A pilot interview was conducted with a qualitative researcher who is a physiotherapist (H.A.) and one patient with a stroke. All of the interviews were undertaken online using the Microsoft Teams platform in the patient’s preferred place or in a virtual clinic in the hospital, which was arranged by the hospital physiotherapist for those patients and carers who have difficulties accessing online. Since Arabic is the mother tongue of both the interviewees and the researcher, the interviews with patients and their carers were conducted in Arabic. As for the physiotherapists, some were interviewed entirely in English, while others used a mix of English and Arabic, depending on their preference.

All the interviews were audio-recorded and transcribed immediately by the first author (B.A.) to increase accuracy. Pseudonyms were applied in this study to ensure the confidentiality of the participants. Any identifying information was removed from each transcript. The first author translated the transcriptions from Arabic to English, and one researcher (H.A.), who is bilingual, reviewed the translated transcripts to ensure the translation reflected the Arabic meaning. Interviews continued until information power was reached [25]. As a result, the research team judged that 22 participants provided sufficient depth and information power to address the study objectives.

### 2.5. Data Analysis

**Figure 1:**
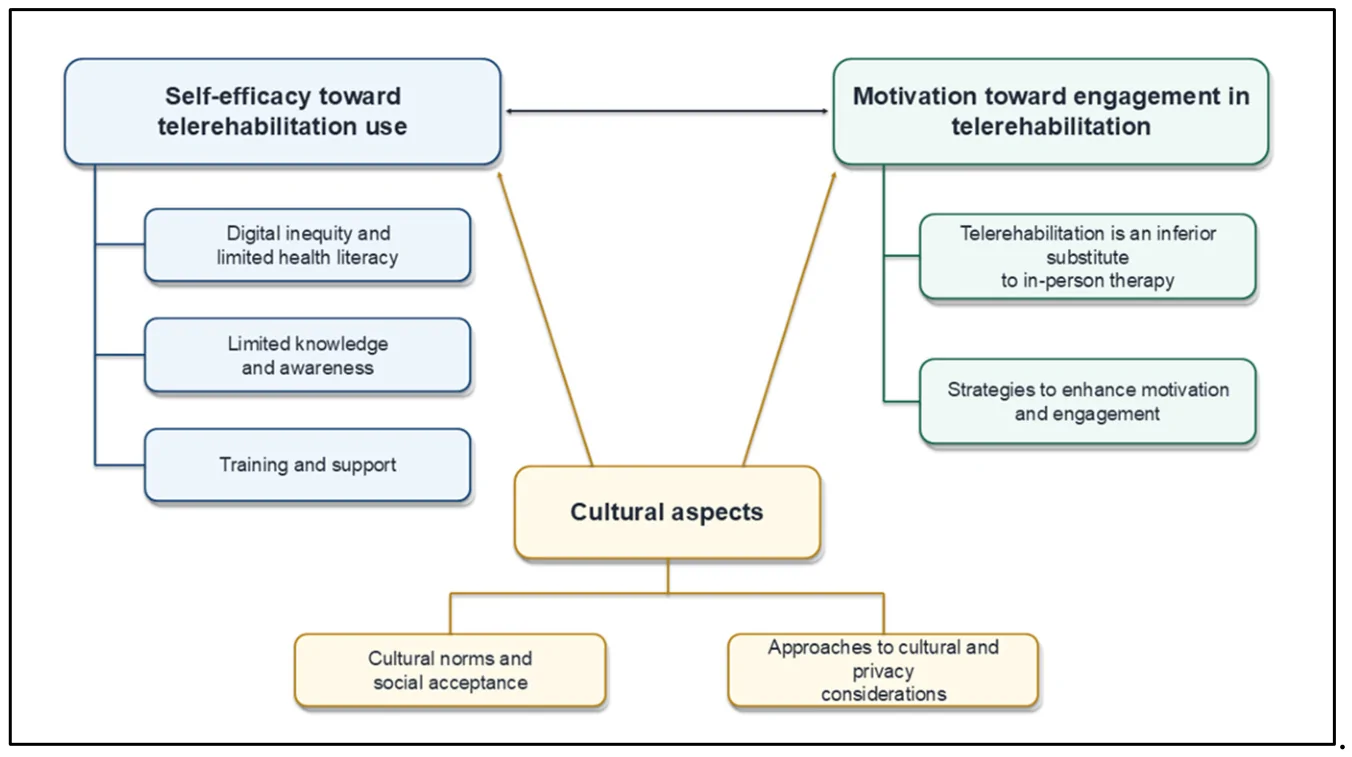
Thematic map.

Reflexive Thematic Analysis (TA) was used to analyse the data as proposed by Braun and Clarke (2021) [26], which includes the following six phases. The first step involved familiarisation with the data by reading and re-reading it multiple times and making notes of thoughts related to the dataset. During this phase, initial codes were identified. Although most patient–carer dyads were interviewed together, the English-translated transcripts were coded and analysed at the individual level. Then, the English-translated transcripts were inductively coded by (B.A.) using NVivo 14 software. Codes were reviewed and discussed by the research team. In reflexive thematic analysis, single-researcher coding is standard and appropriate, while multiple coders may enrich interpretation but are not essential [26]. Following the coding process, the relevant codes were grouped to develop the initial themes. Although participants were coded individually, data from people with stroke, carers, and physiotherapists were analysed together during theme development to identify shared and differing perspectives. Their views were integrated according to their respective roles in rehabilitation, allowing the themes to reflect patterns of meaning across the whole dataset while retaining important stakeholder differences. The developing themes were reviewed in relation to coded extracts and the whole dataset, including interview transcripts. A thematic map (Figure 1) of the analysis was created to visualise how the themes were linked. The final phase, writing up, involves integrating narrative and data extracts and contextualising the analysis within previous literature.

### 2.6. Trustworthiness

Lincoln and Guba’s ‘trustworthiness’ criteria, including credibility, transferability, dependability, and confirmability, are widely applied [27,28]. Although several strategies have been proposed to enhance trustworthiness [29], this study employed purposive sampling, peer debriefing, and triangulation. A purposive sampling strategy was used to capture diverse experiences and perspectives, thereby generating rich data for analysis. Peer debriefing, which involved critical review and feedback from the research team, was another important validation technique employed in this study. In addition, the first author maintained a reflexive approach throughout data collection and analysis by considering how their professional background and positionality may have influenced data collection and interpretation. Finally, triangulation was achieved by engaging various stakeholder groups across two sites.

## 3. Results

### 3.1. Participant Characteristics

A total of 22 participants were interviewed, including ten physiotherapists, six people with stroke and six carers. Most people with stroke and carer dyads chose to be interviewed together, except for one dyad, which was interviewed separately. The interview length ranged from 29 to 55 min.

The physiotherapist participants represented a broad range of experience working with stroke patients across acute, inpatient, and outpatient physiotherapy settings. Their clinical experience ranged from 3 to 16 years (Table 1).

Patients’ ages ranged from 41 to 85 years old, and time post-stroke ranged from 7 weeks to 3 years. All patients had an ischaemic stroke and were male, except for one female patient. The affected side was the left side for all patients except two, whose right side was affected. Cognitive ability (MMSE) showed that all patients had no cognitive impairment (scores ranged from 23 to 29), except for one patient who had mild impairment with a score of 23. Functional ability, measured using the Barthel Index (BI), ranged from 55 to 100, while quality of life, measured by Stroke-Specific Quality of Life (SSQoL) scores, ranged from 125 to 231 (Table 2).

The carers’ ages ranged from 25 to 42 years. Three were male, and three were female. Their relationships with the patients varied and included son, wife, and daughter (Table 3).

### 3.2. Themes and Subthemes

Three themes were developed from the data (Figure 1). The themes were developed across the integrated dataset and reflect patterns of meaning across the perspectives of people with stroke, carers, and physiotherapists. The first theme focused on self-efficacy in the use of telerehabilitation, emphasising the factors that influence it and what is required to enhance self-efficacy and confidence. The second theme related to motivation to use and engage in telerehabilitation was shaped by various barriers and strategies that promote motivation. The final theme explored cultural aspects and approaches that affect self-efficacy and motivation and support the use and implementation of telerehabilitation interventions in clinical practice.

### 3.3. Theme 1: Self-Efficacy Toward Telerehabilitation Use

Self-efficacy, or confidence in one’s ability to perform tasks, is essential for the successful adoption of telerehabilitation. Participants identified barriers such as limited access to digital tools, difficulties understanding health information, and a lack of awareness about telerehabilitation, all of which influence confidence in using telerehabilitation interventions. Adequate technology, knowledge, training, and support are therefore crucial, as increased confidence enhances engagement and improves health outcomes.

#### 3.3.1. Digital Inequity and Limited Health Literacy

Digital inequities and limited health literacy hinder patients’ ability to fully engage in telerehabilitation, contributing to disparities in health outcomes. Several participants reported that in Saudi Arabia, digital inequities significantly limit patients’ access to telerehabilitation. Although healthcare is largely free, the government cannot provide costly devices to all patients, and many families, particularly those in rural or low-income areas, are unable to afford them. Thus, patients without reliable access are less able to benefit from virtual rehabilitation services.

*“The cost of the devices would be a big burden. He wouldn’t be able to afford the equipment and devices.”*

Carer1

*“Because of the equipment they have at the centre. At home it’s hard. If I could read, that would be okay. The therapist at the centre knows the equipment, like the device for the leg, the one for the arm, or the hot packs for ten or fifteen minutes. The therapist comes and helps with the exercises.”*

SP1

Even when technology is available, older adults and individuals with limited literacy may struggle to use it effectively, particularly if they experience cognitive or communication impairments following a severe stroke. These challenges increase the risk of misunderstandings, incorrect exercise performance, and ultimately, widening health disparities in stroke recovery outcomes.

*“There might be technical problems that prevent them from using it, like poor internet service, or they’re in an area with no connectivity. I see these as challenges from the patient’s side. Also, some patients don’t know how to deal with technology. Not everyone in society has that tech literacy.”*

PT10

#### 3.3.2. Limited Knowledge and Awareness

A limited understanding and awareness of telerehabilitation among physiotherapists and patients reduces confidence and acceptance of its use in clinical practice. This, in turn, slows the implementation of telerehabilitation interventions into clinical practice.

Some of the physiotherapist participants have a limited understanding of the telerehabilitation delivery methods, often assuming it simply means phone or video calls (real-time sessions), even though they also record exercises during in-person sessions and share videos and instructions through WhatsApp to support patients in performing exercises at home.

*“I thought it meant live online sessions, like video calls where a patient receives guidance and performs exercises online while being monitored by a therapist remotely.”*

PT2

People with stroke and their carers are similarly unfamiliar with the general concept of telerehabilitation. This low level of awareness creates uncertainty and diminishes confidence in technology-based care, ultimately reducing both patient engagement and physiotherapists’ willingness to adopt telerehabilitation in clinical practice.

*“I’d say its [telerehabilitation] benefit currently doesn’t exceed 35%. I’m referring more to cultural awareness and societal acceptance rather than anything technical. There’s still a belief that ‘this won’t be helpful for me, even if I’m at home,’ and that’s the feedback we get from patients; they want in-person sessions.”*

PT8

#### 3.3.3. Training and Support

Effective and equitable telerehabilitation depends on adequate training, resources, and support for both physiotherapists and patients, along with public awareness and family involvement to ensure quality care and positive outcomes. For telerehabilitation to be effective, physiotherapists require proper training, clear clinical guidelines, reliable infrastructure, and private spaces. Ongoing education, through curriculum integration and professional development, is also essential to sustain competence and confidence in delivering care remotely.

*“We studied physical therapy, but we didn’t study virtual rehab. So, these things require competency.”*

PT9

Patients and caregivers must also be trained, supported, and equipped to use technology effectively at home. This preparation enables them to participate fully in telerehabilitation sessions and to manage exercises and monitoring tasks safely.

*“Technology-based equipment won’t always be available, because I’m not going to a physical place. The whole thing is through a screen, and the device I’m using is either a laptop or a mobile phone. Of course, of course, the patient needs to practice using them.”*

SP3

Several physiotherapists and carers saw that adequate public awareness and active family involvement are critical to providing patients with the supervision and encouragement needed at home. These elements collectively ensure session quality, promote adherence, and contribute to better patient outcomes.

*“I set the time, prepare the space, and get him started. Even our young kids started imitating him, doing the same exercises, which created some excitement and motivation.”*

Carer5

### 3.4. Theme 2: Motivation Toward Engagement in Telerehabilitation

Although some participants perceived telerehabilitation as an inferior alternative to in-person therapy, this view was primarily influenced by factors affecting motivation and adherence. These factors include physical contact, communication, trust, and engagement. However, several strategies have been proposed to mitigate these challenges.

#### 3.4.1. Telerehabilitation Cannot Substitute Face-to-Face Traditional Therapy

Telerehabilitation was perceived as a weaker substitute for face-to-face traditional therapy because it eliminated physical contact and reduced accountability, which undermined the motivation of both physiotherapists and patients toward using and engaging in telerehabilitation.

Physiotherapists reported that the absence of physical contact in telerehabilitation limited accurate assessment, manual interventions, and diagnostic certainty, raising safety concerns for high-risk post-stroke patients.

*“As you know, as PTs, we need to physically touch the patient to properly assess outcomes, like muscle power or range of motion. And if the patient already has movement problems, they need assistance in bed rolling, sitting, standing up, etc. With telerehabilitation, we can only give approximate guidance, but it’s not accurate.”*

PT10

Patients, carers, and physiotherapists emphasised a preference for in-person care, which was viewed as superior for communication, trust, and engagement. Patients highlighted that remote interaction constrained therapeutic outcomes, while additional barriers, including low motivation, insufficient supervision, memory deficits, low mood, and limited space, further reduced adherence to home exercise programs.

*“I’m afraid I’ll do the exercises incorrectly. There’s no way I can perform them with the same precision the doctor showed me. Who will correct me?”*

SP6

These factors impeded recovery and independence and increased healthcare demand in Saudi Arabia, underscoring the value of in-person care and the need for motivational strategies to enhance home exercise delivery.

#### 3.4.2. Strategies to Enhance Motivation and Engagement

Telerehabilitation is effective when implemented after in-person therapy as a complementary approach or when combined with in-person therapy in a Hybrid Model. When it is patient-centred and technologically accessible, it enhances motivation, facilitates continuity of care, and supports adherence to rehabilitation.

Many physiotherapists reported that starting with in-person therapy builds trust, prevents overreliance on technology, and prepares patients for remote care. Telerehabilitation is best introduced after intensive hospital-based rehabilitation, typically in the subacute or chronic phase. While some physiotherapists prefer the subacute stage, others note that applying these interventions in the chronic phase can still sustain hope and motivation.

*“Not acute. I would suggest subacute or chronic. Acute patients need in-person early intervention. They require a multidisciplinary approach with physiotherapy, occupational therapy, speech therapy, and possibly splints. Chronic patients, on the other hand, often just need maintenance therapy to prevent deterioration.”*

PT3

Many of our participants saw that Telerehabilitation is most effective when used as a complementary tool for consultation, monitoring, and follow-up, enabling patients to manage their rehabilitation at home while continuing to receive primary treatment in the hospital.

*“In my situation, remote therapy is fine, but I still think there should be scheduled appointments, like every two or three weeks, where the doctor can check how I’m doing the exercises. They could ask: “How were you doing this exercise?” and then say, “Yes, you’re doing it right, continue like that.”*

SP5

*“It might help, but it shouldn’t be the main approach, not as a primary treatment, just as a supplement. I believe all the therapists should be aligned and agree on the goals.”*

SP6

Physiotherapists recommend emphasising functional goals, particularly gait and balance, to encourage self-management, while simple, user-friendly technologies reduce reliance on caregivers and support independence.

*“Most patients often say the same thing to me: “I want to walk like I used to,” or “I want to return to a normal life.” For example, some patients used to enjoy going to the desert or the farm, and they want to be able to walk there again without a walker or a cane.”*

PT6

Patients are further motivated by practical benefits such as convenience, flexibility in daily routines, and reduced caregiver burden. For those living in rural or remote areas, telerehabilitation also addresses transportation barriers, ensuring continuity of care. Additionally, technologies that provide real-time feedback not only guide and correct movements but also enhance confidence and allow physiotherapists to monitor progress remotely, ultimately strengthening adherence and supporting recovery.

*“Telerehabilitation interventions give him a sense of responsibility to check, apply, and follow up with the doctor. Because sometimes he tells me, “I forgot what exercise the doctor said to do.”*

Carer5

### 3.5. Theme 3: Cultural Aspects

Participants identified several cultural aspects that influence self-efficacy and motivation to use and engage in telerehabilitation interventions, and the overall acceptability of implementing telerehabilitation in the Saudi context. In addition, several approaches were recommended to address these aspects.

#### 3.5.1. Cultural Norms and Social Acceptance

Cultural values, particularly gendered norms, privacy concerns, and social expectations, shaped perceptions of the acceptability of telerehabilitation, regardless of its clinical benefits. In Saudi Arabia, physiotherapists noted that some patients, especially women, felt uncomfortable in attending live sessions and being recorded during the sessions due to cultural, religious, and privacy considerations.

*“There could be barriers, such as patients who don’t want to be filmed, who don’t like having their identity shown. Some patients might have privacy concerns, such as fear about their personal information being exposed, like their name, age, or health status.”*

PT2

While home-based technologies offer convenience, their use is constrained by norms that position the home as a space of family obligations and social interaction. These dynamics disrupt patients’ ability to concentrate on rehabilitation, making clinical settings a more viable context for focused self-care.

*“At home, you know how it is, family, visitors, people coming and going… I can’t focus on my exercises and treatment.”*

SP6

#### 3.5.2. Approaches to Cultural and Privacy Considerations

Telerehabilitation must balance clinical effectiveness with cultural appropriateness to remain both acceptable and practical. To address privacy concerns, physiotherapists reported that they avoid recording entire sessions, instead capturing only key instructions or partial images that are securely stored for clinical use. Patients are reassured about confidentiality and data protection, helping to build trust and compliance.

*“We only record how the exercise is performed, how many repetitions are needed, and other key details to ensure they don’t forget.”*

PT5

Because families play a central role in caregiving after stroke, involving them in remote sessions not only supports patients with memory or comprehension difficulties but also strengthens family involvement and emphasises the importance of self-care for recovery.

## 4. Discussion

This study aimed to gain greater insight into the experiences and perspectives of people with stroke, their carers, and physiotherapists towards the development and implementation of telerehabilitation interventions in physical therapy practice in Saudi Arabia. The study suggested that the successful adoption of telerehabilitation interventions for stroke survivors in Saudi Arabia is constrained by low self-efficacy, limited motivation, and cultural barriers that collectively currently undermine engagement and potentially the clinical impact. Digital inequities, low health and technology literacy, and limited awareness among physiotherapists and people with stroke and their carers diminish confidence in remote rehabilitation and widen disparities. Telerehabilitation is also perceived as an inferior substitute for hands-on therapy, with concerns about safety, communication, supervision, and therapeutic effectiveness reducing motivation and adherence. These challenges are further intensified by cultural norms and privacy concerns, particularly among women and within a busy home environment that limits patients’ capacity to participate fully. While participants recognised the value of hybrid or complementary models that build on in-person therapy, it is clear that without targeted training, structured support, culturally sensitive delivery, and equitable digital access, telerehabilitation risks reinforcing existing gaps rather than improving post-stroke rehabilitation pathways.

Self-efficacy and confidence in the use of telerehabilitation interventions were highlighted as key factors, as digital inequities restrict patients’ access to telerehabilitation interventions and services. These findings are consistent with a recent study by Ouédraogo et al. (2024) on the acceptability of telerehabilitation for individuals with stroke and their caregivers, which identified technical and environmental issues such as internet access, availability of devices, limited space, and lack of human contact [10]. These issues resulted in the cancellation of planned sessions and subsequent planning issues, which may have negative effects on stroke patients’ rehabilitation and therapy intensity [30]. Although technical difficulties are important, they become equity issues when they disproportionately affect certain demographic groups who face challenges accessing technology, such as individuals from lower-income households or those living in rural areas [31]. Overall, these findings underscore that improving self-efficacy in the use of telerehabilitation interventions requires not only addressing technical issues but also addressing digital inequities to ensure equitable access to, and consistent engagement in, rehabilitation for all patients after stroke.

Additionally, our data showed that older and illiterate patients who struggle with using telerehabilitation technologies have, in turn, had their self-efficacy in using technology negatively impacted. This finding was confirmed by Alrushud et al. (2022), who studied the perceptions of physiotherapists delivering telerehabilitation sessions for patients with knee osteoarthritis in Saudi Arabia and found that offering telerehabilitation was not practicable or appropriate for all participants [32]. Similarly, in Nigeria, physiotherapists reported limited patient literacy in using communication technology; therefore, the authors suggested developing an effective method for educating potential patients, as well as raising awareness [33]. Lack of understanding of telerehabilitation delivery methods and suitable training in the use of telerehabilitation among physiotherapists was another barrier affecting self-efficacy. Comparable challenges have been reported in the integration of virtual reality into cancer-supportive care, including insufficient professional training, limited standardised guidance, and accessibility challenges [34]. Training programmes and ongoing education in the field of telerehabilitation were highlighted as potential approaches to overcoming technology-related barriers and enhancing physiotherapists’ competence in delivering physiotherapy services remotely. Similar findings related to training in telerehabilitation practice and topic-specific education were reported in Aljabri et al. (2023), which examined the perspectives of Saudi occupational therapists on telerehabilitation [35]. According to Caughlin et al. (2020), greater adherence requires staff training for new technology as well as clearly defined roles and duties [30]. The acceptance and normalisation of telehealth in the healthcare sector may be enhanced by incorporating these training practices with current educational techniques [30]. Therefore, it is essential to strengthen self-efficacy and confidence in the use of telerehabilitation.

Participants perceived that telerehabilitation would not substitute for in-person traditional therapy, and some of them preferred in-person traditional therapy. These findings are in line with a previous study, which showed that the majority of physiotherapists felt that in-person sessions were more beneficial than telerehabilitation, and many were dissatisfied with the absence of physical contact [32]. This contrasts with the interviews with the managers of physiotherapy departments in Kuwait, which revealed that physiotherapists are generally satisfied with telerehabilitation. However, they also support a hybrid approach, with patients needing to undergo initial assessments in the clinic and then continue remotely for follow-up, due to challenges in using telerehabilitation systems for certain assessments and treatments [36]. Consistent with these findings, most physiotherapists believed that not being able to use ‘hands-on’ techniques would limit their treatment options and make them less effective than face-to-face sessions [37]. Furthermore, some participants reported that therapists’ lack of direct physical contact could lead to inaccurate assessments or treatments [38]. Thus, it has been shown that healthcare providers prefer face-to-face initial appointments to establish patient–clinician rapport and perform objective hands-on techniques [37].

Additional barriers to adherence to the home-exercise program were highlighted by our participants (people with stroke group), particularly their preference for in-person sessions. This is consistent with previous findings that exercise adherence is hindered by several factors, including healthcare practitioners’ lack of emphasis, unfavourable societal influences, a lack of knowledge regarding stroke rehabilitation, and limited access to medical facilities, equipment, or family support [39]. Furthermore, Low motivation and self-efficacy can hinder stroke survivors’ adherence [39]. Therefore, it is important to consider these factors to improve adherence to home-exercise programs after stroke.

In our study, most participants agreed that telerehabilitation interventions offer practical benefits for people with stroke, their carers, and physiotherapists. As reported by Ullah et al. (2021), rehabilitation hospitals in Saudi Arabia are limited in number and concentrated in three major cities: Riyadh, Jeddah, and Dhahran [40]. Travelling for rehabilitation consultations and follow-ups can be costly, result in fewer visits, and create inconvenience for patients and their families [40]. However, participants recommended various strategies to enhance motivation and adherence when using telerehabilitation interventions despite the aforementioned benefits. As in a prior study [38], the majority of participants desired to combine telerehabilitation with in-person visits (hybrid model). A similar result was found in Kuwait, where the majority of physiotherapists thought that telerehabilitation systems could be combined with current conventional systems; however, some physiotherapists preferred to conduct physiotherapy in person [36]. This might be because the field of physiotherapy requires hands-on interventions and a physical presence. These findings, supported by Seron et al. (2021), suggest that hybrid models that combine in-person and telerehabilitation sessions may provide a better balance of accessibility and hands-on care [41]. Therefore, the hybrid model has the potential to significantly improve stroke patients’ rehabilitation care, particularly in situations where resources are inadequate [10]. It would be recommended to use hybrid models to support the continuity of the rehabilitation process.

Some suggested techniques for maximising telehealth implementation involve enhancing technology confidence, technology literacy training, providing access to fast, reliable internet, implementing simple, low-cost technology, promoting self-monitoring, encouraging active engagement, and reviewing patient progress regularly [42]. Telerehabilitation with self-monitoring interventions enhances confidence and sense of security, especially for elderly patients [30]. According to McLean et al. (2013), telerehabilitation therapies can encourage patients to take an active role in their care [43]. For example, wearable sensors can motivate remote interventions while also assessing adherence to study protocols [30]. Incorporating these technologies into telerehabilitation interventions can be an essential consideration [30]. Additionally, people suffering from strokes found that their carers’ support assisted them in using and interacting with telerehabilitation interventions more successfully, particularly in the early phases of stroke recovery when residual stroke effects like issues with fine motor skills, visual impairments, and auditory impairments could make using the technology more difficult [10]. In order to compensate for people with stroke impairments, carers were essential in offering technical support, such as setting up devices and adjusting cameras for improved visibility, as well as fixing any technical problems [10].

Finally, regarding cultural aspects, findings highlighted several cultural barriers, such as gender norms, privacy concerns, and social expectations that influenced the acceptance of telerehabilitation interventions in the Saudi context. These findings are in line with a previous study conducted in Saudi Arabia, which revealed that physiotherapists reported important barriers, including cultural diversity, conservative norms, and differences in accents [32]. Conservative norms, particularly those affecting women, can limit the use of telerehabilitation interventions when they involve recorded videos or video calls [32]. In addition, cultural diversity and the variety of accents spoken across the country may make it difficult for older adults to fully understand exercise instructions [32]. Furthermore, in line with Aljabri et al. (2023), patients in Saudi Arabia may have concerns about privacy or about the use of IT to document certain aspects of their condition or performance [35]. Participants also noted that some patients or carers were worried about their privacy and felt afraid to use cameras and technology when communicating with their healthcare provider [44].

Cultural and religious considerations may also influence the feasibility and acceptability of different telerehabilitation modalities. Religious practices may affect the practical delivery of synchronous telerehabilitation, as daily prayers are observed at specified times and may coincide with healthcare activities, including clinician visits [45]. Therefore, providing flexibility when scheduling synchronous telerehabilitation sessions around prayer times may enhance their cultural acceptability and feasibility. This may be particularly relevant to video conferencing sessions, which require patients and physiotherapists to be available simultaneously. However, asynchronous approaches may provide greater flexibility by allowing patients to engage with rehabilitation at times that are compatible with their religious practices and daily routines.

Family involvement may also act as a facilitator of telerehabilitation rather than solely as a barrier. Although shared family responsibilities may create challenges for home-based rehabilitation, family members can provide supervision, encouragement, and practical support, particularly for patients who have difficulty understanding or remembering rehabilitation instructions. Previous research has similarly shown that family support can help stroke survivors sustain engagement with home-based telerehabilitation [12]. Therefore, understanding and considering cultural factors is important, as this may help identify potential facilitators and inform future research on the use of telerehabilitation for patients in Saudi Arabia [32]. Therefore, understanding the unique cultural factors of patients and carers that influence the effective implementation and acceptance of health technology will support program managers and health care workers in developing and delivering culturally sensitive digital health interventions suited to the local context [44].

Considering the three stakeholder perspectives together suggests that engagement with telerehabilitation is interconnected rather than dependent on the patient alone. People with stroke emphasised the need for reassurance and support, physiotherapists highlighted the importance of clinical oversight and safe delivery, while carers provided practical, motivational, and technological support at home. These perspectives suggest that successful engagement with telerehabilitation may depend on the interaction between professional guidance, family support, and patients’ individual capabilities.

Therefore, to facilitate translation of this study’s findings into intervention development, the identified themes were mapped onto the Behaviour Change Wheel (BCW) [46] and the Theoretical Domains Framework (TDF) [47]. The TDF and BCW did not inform the initial coding or development of themes but were applied after completion of the reflexive thematic analysis to provide a theoretical understanding of the behavioural and contextual factors identified in participants’ accounts. The mapping involved considering the themes, subthemes, and underlying codes against the definitions of the TDF domains, followed by linking the identified domains to relevant BCW intervention functions. Mapping was conducted across the integrated findings rather than separately for each stakeholder group, allowing the perspectives of people with stroke, carers, and physiotherapists to contribute to the same behavioural and contextual determinants where relevant. The mapping identified several relevant TDF domains, including knowledge, skills, beliefs about capabilities, beliefs about consequences, social influences, motivation and goals, and environmental context and resources. These domains were subsequently linked to relevant BCW intervention functions, including education, training, persuasion, enablement, and environmental restructuring (Supplementary Materials Table S1). This post-analysis mapping links the qualitative findings to potential strategies for developing future telerehabilitation interventions in the Saudi context.

### Strengths and Limitations

To our knowledge, this is the first qualitative study to explore in depth the experiences and perspectives of people with stroke, carers, and physiotherapists regarding the implementation of telerehabilitation following stroke in Saudi Arabia. The findings may provide valuable insights, inform clinical practice, and guide future research. The data were collected from SBAHC and KFMC, which are among the largest medical rehabilitation centres in Saudi Arabia and serve patients from different regions across the country.

One limitation of this study is the relative homogeneity of the sample of people with stroke. Five of the six participants were male, although recruitment was not specifically targeted towards men, and all participants had experienced an ischemic stroke. In addition, most participants were in the chronic phase of stroke recovery. Therefore, the findings predominantly reflect the perspectives of men with ischemic stroke at a later stage of recovery and may not fully capture the experiences and perspectives of the broader stroke population in Saudi Arabia. Women, people with haemorrhagic stroke, and those at earlier stages of recovery may have different rehabilitation needs, experiences, and perspectives towards telerehabilitation. In particular, people at earlier stages of recovery may have different levels of dependency and require greater support from physiotherapists or carers, which could influence their perceptions of and readiness for telerehabilitation. Therefore, the transferability of the findings to these groups should be considered with caution. Future research should consider greater diversity in terms of sex, stroke type, and stage of recovery to explore a broader range of perspectives towards telerehabilitation.

The study also excluded patients with significant cognitive impairments and communicative difficulties; therefore, the findings may not capture the perspectives and needs of these groups. In addition, most people with stroke were interviewed jointly with their carers. Although responses from people with stroke and carers were clearly distinguished during data collection and analysis, their joint participation may have influenced how individual perspectives were expressed. Furthermore, online interviews were conducted; the lack of face-to-face interviews may have resulted in the absence of body language and facial expression cues [48]. Additionally, member checking was not conducted as it is not considered an essential quality practice in reflexive thematic analysis. Finally, the study’s findings are limited to the Saudi Arabian context and may not be generalizable to other settings.

## 5. Conclusions

The successful adoption of telerehabilitation interventions for people with stroke in Saudi Arabia depends on two interconnected factors: self-efficacy and motivation, which are influenced by cultural aspects. Barriers such as digital inequity, limited knowledge and awareness, lack of training, cultural norms, and a strong preference for in-person therapy can collectively affect them. However, our findings indicate that, when supported by adequate training, family involvement, culturally sensitive practices, and hybrid models that combine in-person and remote care, telerehabilitation has the potential to enhance access, sustain motivation, and support continuity of rehabilitation in Saudi Arabia. To move towards implementation, future intervention development should prioritise simple and accessible technology, structured training for physiotherapists, patients, and carers, appropriate family involvement, and flexible hybrid delivery that considers patients’ preferences and cultural needs. These components should first be evaluated through feasibility studies in the Saudi context before wider implementation.

## Figures and Tables

**Table 1:** Characteristics of the sample (physiotherapists, *n* = 10).

Code PT	Gender	Profession (Clinical/Academic Physiotherapist)	Years of Experience in Neurorehabilitation	Primary Location (Acute Care, Inpatient, Outpatient)
PT1	Male	Clinical	3 years	Outpatient
PT2	Female	Clinical	7 years	Outpatient
PT3	Female	Clinical	16 years	Inpatient
PT4	Male	Clinical	6 years	Outpatient
PT5	Female	Clinical	6 years	Outpatient
PT6	Male	Clinical	3 years	Outpatient
PT7	Female	Clinical	5 years	Outpatient
PT8	Male	Clinical	7 years	Outpatient
PT9	Female	Clinical	6 years	Acute care
PT10	Male	Clinical	9 years	Inpatient

(PT): Physiotherapist.

**Table 2:** Characteristics of the sample (people with stroke; *n* = 6).

Code SP	Gender	Age	Type of Stroke (Ischemic/ Haemorrhagic)	Time Post-Stroke	Affected Side (RT, LT)	Cognitive Ability	Functional Ability	Health-Related Quality of Life
						(MMSE) *	(BI) *	(SSQoL) *
SP1	Male	85	Ischemic	3 years	RT	29	55	148
SP2	Male	65	Ischemic	1 year and 7 months	LT	27	60	133
SP3	Male	51	Ischemic	12 months	LT	29	100	193
SP4	Male	81	Ischemic	2 years	RT	23	65	167
SP5	Male	41	Ischemic	7 weeks	LT	28	100	231
SP6	Female	50	Ischemic	2 years	LT	29	70	125

* (MMSE): Mini-Mental State Examination; * (BI): Barthel Index; * (SSQoL): Stroke Specific Quality of Life (SSQoL); (SP): Stroke Patient.

**Table 3:** Characteristics of the sample (Carers; *n* = 6).

Code Carer	Gender	Age	Relationship to Patients	Individual Interview or Together
Carer 1	Male	25	Son	Together
Carer 2	Female	42	Wife	Together
Carer 3	Male	29	Son	Individual
Carer 4	Male	26	Son	Together
Carer 5	Female	36	Wife	Together
Carer 6	Female	26	Daughter	Together

## Data Availability

The data presented in this study are available upon request from the corresponding author due to participants’ privacy and ethical requirements.

## Supplementary Materials

The following supporting information can be downloaded at https://www.mdpi.com/article/10.3390/healthcare14172877/s1. (COREQ) checklist, UABCC, and Table S1. Post-analysis mapping of themes and subthemes to TDF domains and BCW intervention functions.

## Appendix A. Semi-Structured Interview Guide

**Table A1:** Physiotherapists.

	Questions	Prompts
1	Can you tell me about your experience throughout your clinical practice?	Number of years Inpatients/outpatients Neurological conditions Rehabilitation of people with stroke The current practice in medical rehabilitation (for acute, subacute, and chronic) Number of stroke cases every week. The most cases you deal with. (acute, subacute, chronic)
2	Can you tell me about your experience of using a telerehabilitation program after a stroke?	If yes? How many years? During COVID or after? If no, what do you think about using telerehabilitation? The best technology? Examples. Why? How can we give patients intervention through this technology? e.g., Exercises. Outcomes For which phase? Factors that should be considered for content and delivery.
3	What do you think would facilitate the implementation of telerehabilitation in physical therapy practice in SA?	Facilitators
4	What do you think are the barriers and difficulties to implementing telerehabilitation in physical therapy practice in SA?	Barriers

**Table A2:** People with stroke.

	Questions	Prompts
1	Tell me about yourself. حدثني عن نفسك	Age Work children
2	Tell me about your condition. أخبرني عن حالتك	When? How? Affected side. Functional ability.
3	Tell me about your rehabilitation sessions. أخبرني عن جلسات التأهيل الخاصة بك	In-person/or online How many sessions for inpatients/outpatients? Outcome: UL motor function, gait, LL motor function, balance. Home exercises program/barriers.
4	The current practice in different countries is to use telerehabilitation interventions, which means the delivery of physiotherapy services such as Exercises via information and communication technology, including videoconferencing, VR, telephone, and mobile applications, and sensors. What do you think about this? تعتمد الممارسات الحالية في مختلف البلدان على استخدام تدخلات التأهيل عن بُعد، والتي تعني تقديم خدمات العلاج الطبيعي مثل التمارين عبر تكنولوجيا المعلومات والاتصالات، بما في ذلك مكالمات الفيديو، والهاتف، وتطبيقات الهاتف المحمول، والمستشعرات. ما رأيك في هذا؟	Experience. Perspectives Benefits Outcomes. Factors that should be considered for content and delivery.
5	What do you think would facilitate the implementation of telerehabilitation in physical therapy practice in SA? ما الذي تعتقد أنه سيسهل تطبيق العلاج عن بعد في ممارسة العلاج الطبيعي في المملكة العربية السعودية؟	Facilitators/needs
6	What do you think are the barriers and difficulties to implementing telerehabilitation in physical therapy practice in SA? ما هي برأيك العوائق والصعوبات التي تواجه تطبيق العلاج عن بعد في مجال العلاج الطبيعي في المملكة العربية السعودية؟	Barriers

**Table A3:** Carers.

	Questions	Prompts
1	Tell me about yourself. حدثني عن نفسك	Age Work Relationship to the patient.
2	Tell me about your experience as a carer for patients with stroke. أخبرني عن تجربتك كمقدم رعاية لمرضى السكتة الدماغية	How did she/he receive her/his rehabilitation session? In-person/or online. How many sessions? (inpatients/outpatients). Outcome: UL motor function, gait, LL motor function, balance? Tell me about your support as a carer. Barriers you face during this period.
3	The current practice in different countries is to use telerehabilitation interventions which means the delivery of physiotherapy services such as Exercises via information and communication technology, including videoconferencing, telephone, mobile applications, and sensors. What do you think about this? تعتمد الممارسات الحالية في مختلف البلدان على استخدام تدخلات التأهيل عن بُعد، والتي تعني تقديم خدمات العلاج الطبيعي مثل التمارين عبر تكنولوجيا المعلومات والاتصالات، بما في ذلك مكالمات الفيديو، والهاتف، وتطبيقات الهاتف المحمول، والمستشعرات. ما رأيك في هذا؟	Experience. As a carer, how would telerehabilitation impact your role with your patient? Benefits Outcomes Factors that should be considered for content and delivery.
4	What do you think would facilitate the implementation of telerehabilitation in physical therapy practice in SA? ما الذي تعتقد أنه سيسهل تطبيق العلاج عن بعد في ممارسة العلاج الطبيعي في المملكة العربية السعودية؟	Facilitators/needs
5	What do you think are the barriers and difficulties to implementing telerehabilitation in physical therapy practice in SA? ما هي برأيك العوائق والصعوبات التي تواجه تطبيق العلاج عن بعد في مجال العلاج الطبيعي في المملكة العربية السعودية؟	Barriers
