# Supplementary material for: Experiences and Perspectives of People with Stroke, Carers, and Physiotherapists Toward Telerehabilitation Implementation Following Stroke in Saudi Arabia: A Qualitative Study
Source: Healthcare (Basel). 2026 Sep 7;14(17):2877. doi: 10.3390/healthcare14172877 (PMC13565887; doi:10.3390/healthcare14172877)
Supplement: Supplementary file 1 [file healthcare-14-02877-s001.zip › Supplementary Material- UABCC.pdf]

The University of California, San Diego Brief Assessment of Capacity to Consent (UBACC)[1].

The UBACC is intended to help investigators identify research participants who warrant more thorough decisional capacity assessment and/or remediation efforts prior to enrolment.

Participants need to achieve a score of 7 or 8 on the eight decisional capacity questionnaire related to the content of the consent form.

Question	Score
**1. What is the purpose of the study that was just described to you? ما هو هدف الدراسة التي تم وصفها لك**<br><br>a) To develop new medication لتطوير أدوية جديدة<br>b) To explore experiences & perspectives of patients with stroke and their carers toward using technologies to deliver rehabilitation services لاستكشاف تجارب وتوقعات المرضى الذين يعانون من السكتة الدماغية ومقدمي الرعاية لهم تجاه استخدام التكنولوجيا لتقديم خدمات إعادة التأهيل<br><br>**Response (**2= study explore experiences & perspectives of patients with stroke and their carers toward using technologies to deliver rehabilitation services)	Score<br>0<br>1<br>2
**2. What makes you want to consider participating in this study? ما الذي يجعلك تفكر للمشاركة في هذه الدراسة؟**<br>a) The study will lead to the development of new interventions. ستؤدي الدراسة الى تطوير تدخلات جديدة<br>b) Research is important because it can help other people. البحث مهم لأنه يمكن أن يساعد الآخرين<br><br>**Response** (2 = the study will lead to the development of new intervention)	Score<br>0<br>1<br>2
**3. Do you believe this is primarily research or primarily treatment? هل تعتقد أن هذا البحث هو في الأساس دراسة بحثية أم علاج؟**<br>a) Research. البحث<br>b) Treatment. العلاج<br><br>**Response** (2 = Research)	Score<br>0<br>1<br>2
**4. Do you have to be in this study if you do not want to participate? هل يجب أن تكون في هذه الدراسة إذا كنت لا ترغب في المشاركة؟**<br>a) Yes نعم<br>b) No لا<br><br>**Response** (2 = No)	Score<br>0<br>1<br>2
**5. If you withdraw from this study, will you still be able to receive regular treatment? إذا انسحبت من هذه الدراسة، هل ستظل قادراً على تلقي العلاج العادي؟**<br>a) Yes نعم<br>b) No لا<br><br>**Response** (2 = Yes)	Score<br>0<br>1<br>2

**6. If you participate in this study, what are some of the things that you will be asked to do? إذا شاركتا في هذه الدراسة، ماهي الأشياء التي سيطلب منك القيام بها؟**<br>a) respond to the questions in the interview. الرد على الأسئلة في المقابلة<br>b) Do physical activity every day. القيام بنشاط بدني كل يوم<br><br>**Response (**2 = respond to the questions in the interview)	Score<br>0<br>1<br>2
**7. Please describe some of the risks or discomforts that people may experience if they participate in this study. يرجى وصف بعض المخاطر التي قد يواجهها الأشخاص إذا شاركوا في هذه الدراسة**<br>a) This study tests a new drug; that may give you unpleasant symptoms هذه الدراسة تختبر دواء جديد ؛ قد يسبب لك أعراضاً مزعجة<br>b) This study answers questions related to your experiences and had no risks. هذه الدراسة تجيب على أسئلة تتعلق بتجاربك وليس لها أي مخاطر<br><br>**Response** (2 = No risks)	Score<br>0<br>1<br>2
**8. Please describe some of the possible benefits of this study. يرجى وصف بعض الفوائد المحتملة لهذه الدراسة**<br>a) Your participation will improve the future clinical and research work in stroke rehabilitation. ستعمل مشاركتك على تحسين العمل البحثي والسريري المستقبلي في إعادة تأهيل السكتة الدماغية<br>b) There are no personal benefits from participating in this study لا توجد فوائد شخصية من المشاركة في هذه الدراسة<br><br>**Response** (2= Your participation will improve future clinical and research work)	Score<br>0<br>1<br>2

1. Jeste, D.V.; Palmer, B.W.; Appelbaum, P.S.; Golshan, S.; Glorioso, D.; Dunn, L.B.; Kim, K.; Meeks, T.; Kraemer, H.C. A new brief instrument for assessing decisional capacity for clinical research. *Archives of general psychiatry* **2007**, *64*, 966-974.
