# Supplementary material for: Experiences and Perspectives of People with Stroke, Carers, and Physiotherapists Toward Telerehabilitation Implementation Following Stroke in Saudi Arabia: A Qualitative Study
Source: Healthcare (Basel). 2026 Sep 7;14(17):2877. doi: 10.3390/healthcare14172877 (PMC13565887; doi:10.3390/healthcare14172877)
Supplement: Supplementary file 1 [file healthcare-14-02877-s001.zip › Supplementary Material-Post-analysis mapping of themes and subthemes to TDF domains and BCW.pdf]

Table S1. Post-analysis mapping of themes and subthemes to TDF domains and BCW intervention functions

Theme	Subtheme	Relevant TDF domain(s)	BCW intervention function(s)	Brief explanation
1. Self-Efficacy Toward Telerehabilitation Use	Digital inequity and limited health literacy	Environmental context and resources; Skills	Environmental restructuring; Training; Enablement	Limited access to devices/internet and difficulties using technology can reduce patients' capability and opportunity to engage in telerehabilitation.
	Limited knowledge and awareness	Knowledge	Education	Limited awareness and understanding of telerehabilitation among patients and physiotherapists can reduce confidence and acceptance; education and awareness are therefore required.
	Training and support	Skills; Social influences	Training; Enablement	Patients and physiotherapists require appropriate training, while family and professional support can facilitate technology use and rehabilitation at home.
2. Motivation Toward Engagement in Telerehabilitation	Telerehabilitation cannot substitute for face-to-face traditional therapy	Beliefs about consequences; Beliefs about capabilities	Persuasion; Enablement	Concerns about physical contact, assessment accuracy, safety, supervision and effectiveness contribute to perceptions of telerehabilitation as inferior to face-to-face care.
	Strategies to enhance motivation and engagement	Motivation and goals; Beliefs about capabilities	Persuasion; Enablement	Hybrid delivery, functional goal setting, real-time feedback and continued professional monitoring may strengthen confidence, motivation and engagement.
3. Cultural Aspects	Cultural norms and social acceptance	Social influences; Environmental context and resources	Enablement; Environmental restructuring	Gender norms, privacy concerns, family obligations and the home environment influence the acceptability and practical use of telerehabilitation.
	Approaches to cultural and privacy considerations	Social influences; Environmental context and resources	Enablement; Environmental restructuring	Culturally sensitive delivery, privacy protection and family involvement may reduce contextual and social barriers to implementation.

Note. TDF = Theoretical Domains Framework; BCW = Behaviour Change Wheel. The mapping was undertaken after completion of the reflexive thematic analysis and did not inform initial coding or theme development.
